# Variation in the Large-Scale Organization of Gene Expression Levels in the Hippocampus Relates to Stable Epigenetic Variability in Behavior

**DOI:** 10.1371/journal.pone.0003344

**Published:** 2008-10-06

**Authors:** Mark D. Alter, Daniel B. Rubin, Keri Ramsey, Rebecca Halpern, Dietrich A. Stephan, L. F. Abbott, Rene Hen

**Affiliations:** 1 Department of Psychiatry, Columbia University, New York, New York, United States of America; 2 Department of Neuroscience, Columbia University, New York, New York, United States of America; 3 Department of Neuroscience and Division of Theoretical Neuroscience, Columbia University, New York, New York, United States of America; 4 Neurogenomics Division, Translational Genomics Research Institute, Phoenix, Arizona, United States of America; Minnesota State University Mankato, United States of America

## Abstract

**Background:**

Despite sharing the same genes, identical twins demonstrate substantial variability in behavioral traits and in their risk for disease. Epigenetic factors–DNA and chromatin modifications that affect levels of gene expression without affecting the DNA sequence–are thought to be important in establishing this variability. Epigenetically-mediated differences in the levels of gene expression that are associated with individual variability traditionally are thought to occur only in a gene-specific manner. We challenge this idea by exploring the large-scale organizational patterns of gene expression in an epigenetic model of behavioral variability.

**Methodology/Findings:**

To study the effects of epigenetic influences on behavioral variability, we examine gene expression in genetically identical mice. Using a novel approach to microarray analysis, we show that variability in the large-scale organization of gene expression levels, rather than differences in the expression levels of specific genes, is associated with individual differences in behavior. Specifically, increased activity in the open field is associated with increased variance of log-transformed measures of gene expression in the hippocampus, a brain region involved in open field activity. Early life experience that increases adult activity in the open field also similarly modifies the variance of gene expression levels. The same association of the variance of gene expression levels with behavioral variability is found with levels of gene expression in the hippocampus of genetically heterogeneous outbred populations of mice, suggesting that variation in the large-scale organization of gene expression levels may also be relevant to phenotypic differences in outbred populations such as humans. We find that the increased variance in gene expression levels is attributable to an increasing separation of several large, log-normally distributed families of gene expression levels. We also show that the presence of these multiple log-normal distributions of gene expression levels is a universal characteristic of gene expression in eurkaryotes. We use data from the MicroArray Quality Control Project (MAQC) to demonstrate that our method is robust and that it reliably detects biological differences in the large-scale organization of gene expression levels.

**Conclusions:**

Our results contrast with the traditional belief that epigenetic effects on gene expression occur only at the level of specific genes and suggest instead that the large-scale organization of gene expression levels provides important insights into the relationship of gene expression with behavioral variability. Understanding the epigenetic, genetic, and environmental factors that regulate the large-scale organization of gene expression levels, and how changes in this large-scale organization influences brain development and behavior will be a major future challenge in the field of behavioral genomics.

## Introduction

Although genetically identical twins in humans and other organisms are strikingly similar in appearance, they are paradoxically discordant for many important phenotypes and complex diseases [1], [2]. These differences are traditionally attributed to environmental factors even though studies of identical twins reared together or apart do not support this explanation [3], [4]. Increasing evidence points to the importance of epigenetic factors–DNA and chromatin modifications that affect the levels of gene expression without affecting DNA sequence–in the generation of these phenotypic differences. Experiments in genetically identical mice, for example, suggest the importance of epigenetic influences on individual variability in many phenotypic traits. Monozygotic twin mice (genetically identical mice that are generated from a single zygote by dividing the blastocyst at the 8-cell stage to produce 2 viable embryos) are significantly more alike for multiple complex traits than are dizygotic twin mice (genetically identical mice obtained from different zygotes) [5]. Subsequent work demonstrates that, in addition to the epigenetic differences that are present at the earliest stages of development, epigenetic differences also are affected by experience during critical periods of development. In rodents, for example, variations in maternal care during the early postnatal period produces modifications of chromatin and demethylation of DNA within offspring brains which in turn are associated with stable changes in gene expression, brain development, and behavior [6]. Thus, both inherited and environmental factors produce epigenetic modifications that, in turn, influence gene expression and phenotypic variation.

Attempts have been made to study patterns of gene expression that are associated with variability in behavior that is under epigenetic control. Microarray studies suggest that epigenetic factors can have extensive effects on levels of gene expression [7]. Traditional microarray analysis, however, is not well suited to the study of the large-scale organization of gene expression levels because traditional approaches to microarray analysis use normalization procedures that minimize global differences in gene expression so as to improve the detection of specific changes in gene expression levels that exceed a pre-specified threshold relative to the expression levels of other genes. More recently, a global approach to microarray analysis was used to study the large-scale organization of gene expression levels [8], [9]. We reasoned that a global microarray-based approach, in contrast to a traditional gene-centered one, might reveal changes in the large-scale organization of gene expression levels that are associated with epigenetic influences on phenotypic variability in genetically identical mice. Herein we show that variability in the large-scale organization of gene expression levels in the hippocampus in genetically identical mice is associated with individual differences in the activity of mice in the open field and that an early developmental intervention that modifies activity in the open field of adult mice also modifies their large-scale organization of gene expression levels. We also report similar findings in outbred mice that were selected for extremes of contextual fear suggesting that our findings may be relevant to outbred populations such as humans.

## Results

### Variance in log-transformed levels of gene expression is associated with activity in the open field within inbred mice

To examine epigenetic influences on the large-scale organization of gene expression levels, we studied the relationship of gene expression organization in the hippocampus with stable individual differences in activity in the open field in genetically identical mice. As an initial measure of gene expression organization we examined overall variance of log-transformed gene expression levels. Gene expression levels were log-transformed because gene expression levels do not follow a normal distribution. Gene expression levels are highly skewed such that a majority of genes are expressed at low levels while a relatively small number of highly expressed genes account for the majority of total gene expression. Variance of log-transformed gene expression levels is a measure of the spread of the data distribution. Increased variance indicates that there is a larger difference between gene expression levels of high and low expressed genes and reflects an increased range of gene expression. Our studies suggest that changes in the variance of log-transformed gene expression levels may have functional relevance.

**Figure 1:**
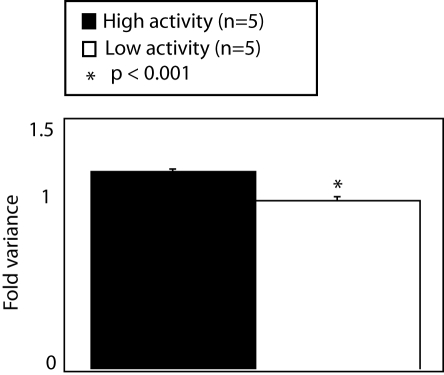
Variance of log-transformed gene expression levels in the hippocampus is associated with individual differences in activity in the open field. Gene expression was log-transformed to reduce normally distributed noise and because of a recognized skewing of gene expression data to low expression values. Variance of log-transformed gene expression levels was significantly increased in mice selected for stable high activity in the open field.

Rodents are known to exhibit stable individual variability in behavior across the lifespan [10], [11]. To focus on individual variability that is associated with epigenetic factors, we used inbred mice (genetically identical) born on the same day and raised under identical conditions. Stability of activity in the open field was identified with repeated testing of mice in the open-field paradigm. A composite behavioral score (see methods) was used to highlight the stability of behavior. Animals separated by composite behavioral scores demonstrated significant differences in all open field measures (supplemental figure S1). Variance of log-transformed gene expression levels in the hippocampus, a region known to influence activity in the open field, of the top and bottom 25% of animals sorted by composite behavioral scores was significantly increased in more active mice (figure 1).

### Postnatal handling modifies adult activity in the open field and the large-scale organization of gene expression levels

**Figure 2:**
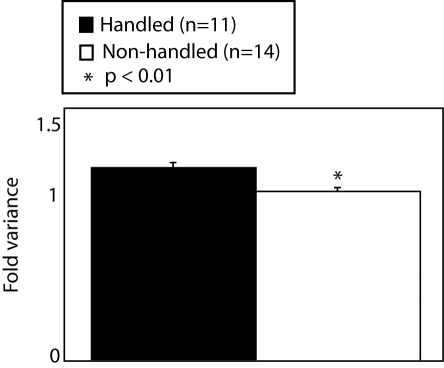
Variance of log-transformed gene expression levels in the CA1 of the hippocampus is affected by postnatal handling. Postnatal handling resulted in an increased variance of log-transformed gene expression levels in the same direction and magnitude as in high activity mice. Changes in gene expression organization were accompanied by increased activity in the open field (supplemental figure S2) and increased activity in the light compartment in the light-dark paradigm (supplemental figure S3).

Because the large-scale organization of gene expression levels was associated with phenotypic differences in activity in the open field, we predicted that a developmental intervention that modifies activity in the open field would lead to a change in the large-scale organization of gene expression levels. To test this hypothesis, we exposed newborn mice to postnatal handing, a well-described developmental intervention in rodents that, through epigenetic mechanisms, increases activity of adult rodents in the open field [12]. As previously reported [12], postnatal handling significantly increased adult activity in the open field as measured by composite behavioral scores and individual measures of activity in the open field (supplemental figure S2). In the light-dark paradigm, we also found increased activity in the light compartment but no increase in overall activity (supplemental figure S3). Increased activity in the light compartment for handled mice suggests an increase in exploratory drive or a decreased fear of brightly lit areas. This pattern of behavior is often interpreted as a decrease in anxiety. We found that, in the CA1 region of the hippocampus, a region previously linked to the epigenetic effects of early life experience on the glucocorticoid receptor gene [13], the more active handled mice had a significant increase in the variance of log-transformed gene expression levels (figure 2). Strikingly, the change in the variance in log-transformed gene expression levels in handled mice was similar in direction and magnitude as when we compared high to low activity mice.

### Variance of log-transformed gene expression levels is associated with behavior in outbred mice

To further evaluate the robustness of our findings, we examined two publically available data sets from the Gene Expression Omnibus public repository. Because we found that the large-scale organization of gene expression levels in the hippocampus was associated with behavioral variability in our studies, we predicted that studies of gene expression in the hippocampus performed by others and available through the Gene Expression Omnibus public repository would exhibit a similar association of the large-scale organization of gene expression levels with behavioral variability. We searched for data sets that met the following criteria: 1) 430_2.0 mouse microarray platform, 2) conducted at a NIH Neuroscience Microarray Consortium site (arrayconsortium.tgen.org), 3) analysis of gene expression in the hippocampus, and 4) characterization of hippocampus-related behavior. These criteria assured that similar microarray techniques were used, that high quality control standards were met, that the brain regions remained constant, and that gene expression patterns could be compared to behavioral variability. Importantly, we have found that measurements of variance in log-transformed levels of gene expression are sensitive to both biological and technical factors (see below). Therefore, it is important when the large-scale organization of gene expression levels is examined that samples be processed in parallel, as is always done by microarray consortium sites. We found two data sets that met our criteria and were generated from a lab studying patterns of gene expression in genetically heterogeneous mice that were outbred and selected for extremes of contextual fear conditioning behavior, a well-studied hippocampus-related behavior [14], [15]. (see supplemental figure S4 for a schematic of breeding strategy).

In these studies, an anxious (DBA/2J or A/J) and non-anxious (C57BL/6J) strain of mouse were crossed and intercrossed to obtain F2 progeny. F2 progeny were selected on contextual fear. The top and bottom approximately 10% of offspring by contextual freezing scores were bred to generate the next generation. This was continued for 4–5 generations. Mice selected in this manner not only differed in contextual freezing, but in the case of the DBA/2J×C57BL/6J cross and short-term selection, mice also differed in the elevated plus maze and open field paradigms with low freezing mice displaying increased time in the center of the open field and open arms of the elevated plus maze [14]. The pattern of behavior in open field activity for low freezing mice was similar to the high activity and handled groups of mice in our experiments. For each data set, the authors looked at gene expression levels in the hippocampus and the amygdala of mice selected for high and low freezing. Mice used for microarray studies were not subjected to behavioral testing. The use of behaviorally naïve mice rules out the possibility that was not addressed in our experiments that effects of behavioral testing on levels of gene expression might account for differences in the large-scale organization of gene expression levels that we found between groups. Because the authors examined gene expression in both the hippocampus and the amygdala, we were also able to address the question of regional specificity of differences in the large-scale organization of gene expression levels that are associated with behavioral variability.

**Figure 3:**
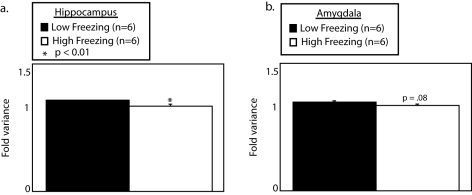
Variance of log-transformed gene expression levels in the hippocampus but not the amygdala is associated with contextual fear and present in genetically heterogeneous mice. Gene expression data was downloaded from the Gene Expression Omnibus public repository (GSE4034 & GSE4035 (supplemental figure S4)). Mice for these experiments were selected over multiple generations after an inter-strain cross of a high anxiety (DBA or A/J) and low anxiety (C57/B6) strain of mice. We found that variance of log-transformed gene expression levels in the hippocampus but not amygdala was significantly increased in low freezing mice. In this study low freezing mice were found to have increased activity in the open-field similar to high activity and handled mice [14].

We found, as was predicted by the pattern of behavior in the open field, that low freezing mice had increased variance in log-transformed gene expression levels in the hippocampus (figure 3). Interestingly, when gene expression in the amygdala was examined, significant differences in the variance were not found suggesting that the association of gene expression organization with contextual freezing was limited to or at least more extensively affected in the hippocampus than in the amygdala. The results were similar in a second experiment performed by the same lab (supplemental figure S5). The authors of that study noted and we agree that one possible explanation for less pronounced effects on measured levels of gene expression in the amygdala is that the amygdala is a heterogeneous structure with multiple nuclei. Therefore, it is possible that the heterogeneity of the amygdala may have obscured a difference in the large-scale organization of gene expression levels.

### Increased variance is associated with an increased separation of 3 large log-normally distributed families of gene expression levels

**Figure 4:**
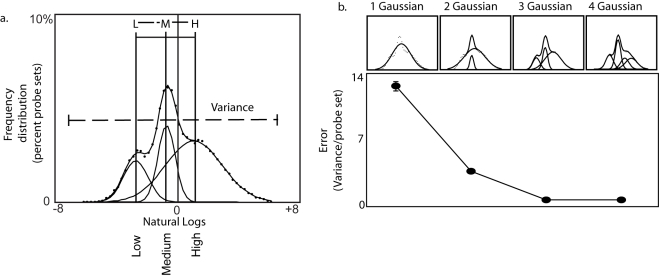
Global gene expression is described by three log-normal distributions. We found that the distribution log-transformed gene expression levels can be described by three log-normal curves. Three normal distributions were fit to log-transformed Affymetrix micro-array data that was processed and summarized using MAS 5.0. The fit error in all cases was minimal and could not be improved significantly by adding additional log-normal curves, whereas trying to fit data to one or two log-normal distributions significantly increased error. Matlab fitting algorithms perform well across a wide range of distribution patterns (supplemental figure S6).

To better understand the finding of increased variance of log-transformed gene expression levels in the hippocampus within more active mice, we took a closer look at the overall distribution pattern of gene expression levels. To our surprise, we found at the global level that gene expression levels were characterized by 3 log-normal distributions oriented from low to high expression levels (figure 4). This is consistent with reports that describe the existence of 3 abundance classes of mRNAs based on the dynamics of labeled cDNA-to-total mRNA hybridization, a global analysis technique used before microarray technology became available [16], [17]. Log-normal distributions arise in the presence of multiple, independent, and multiplicative forces. They are common throughout nature [18]. The presence of 3 log-normal distributions of gene expression levels, suggested that genes belonging to a given distribution would share regulatory mechanisms in common, and, therefore, might move together as a group relative to other distributions. We hypothesized that the independent movement of individual log-normal distributions of gene expression levels might be associated with changes in the variance of log-transformed gene expression levels that were associated with epigenetically mediated variability in activity of mice in the open field.

**Figure 5:**
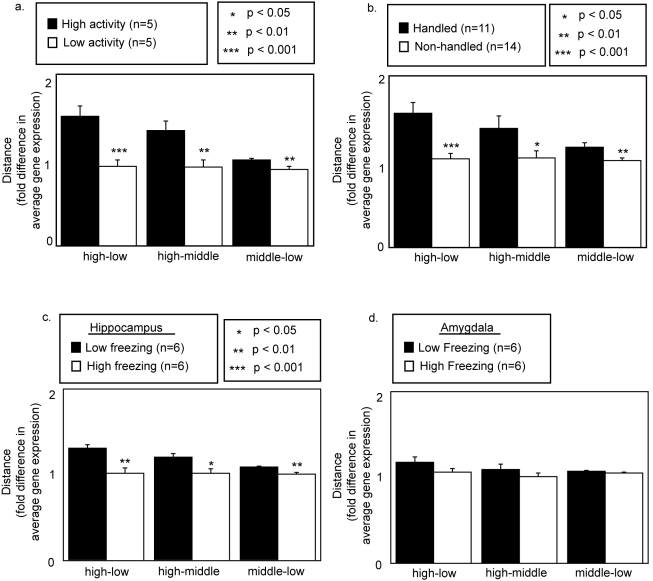
Increased variance in gene expression in more active mice relates to a moving apart of log-normal distributions. We find, in all cases where variance is increased, that a corresponding increase in the distance between log-normal distributions is present. The distance is measured between peaks in natural logs. We present the data normalized to the less active group of animals and converted into linear fold change of expression levels. For example, if the distance between the high and low expression groups of genes was 1 natural log greater in more active animals, then the difference would be the natural exponent taken to the power of 1 or 2.718. This would mean that when compared to the low expression group of genes that the high expression group of genes is up-regulated on average 2.718-fold. Significant differences in separation were found between the low and middle, middle and high, and high and low distributions in all cases. Importantly, significant differences in separation of distributions were found in the hippocampus but not in the amygdala between high and low freezing mice. This is consistent with the findings for the variance (figure 3).

To examine the relative expression levels of different log-normal distributions we used curve fitting tools in Matlab to fit 3 normal distributions to log-transformed data. By back-transforming the distances between the peaks of log-normal distributions, we converted relative distances between peaks of log-normal distributions into linear fold differences in the average log expression levels of genes belonging to the gene expression family. We found in all cases where the variance was increased that this was accompanied by an increased separation of log-normal distributions of gene expression levels. The increased variance, thus, was not simply explained by a widening of individual distributions but was associated with independent relative changes in the average log expression levels of 3 large families of gene expression. The changes in the average levels of gene expression that were associated with an increased separation of log-normal distributions were not small. When referenced to the low expression family of gene expression, the high expression family of gene expression was up-regulated on average by greater than 1.5 fold in mice with high vs. low activity in the open field or mice that were handled vs. those that were raised under standard conditions (figure 5). Interestingly, as was suggested by the measurement of variance in gene expression levels in high and low freezing mice, we only saw significant changes in the separation of log-normal gene expression distributions in the hippocampus and not in the amygdala. This suggests that global changes in the large-scale organization of gene expression levels that are associated with behavioral variability may be specific to certain brain regions and not to others.

### The presence of multiple log-normally distributed gene expression families is a universal feature of eukaryotic gene expression

**Figure 6:**
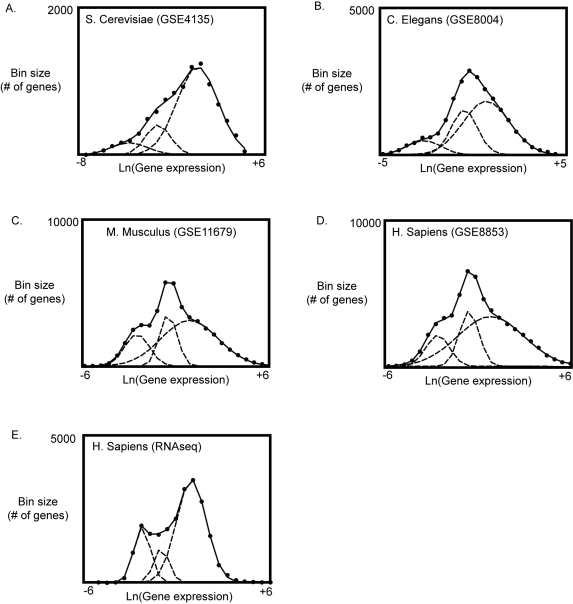
The presence of 3 log-normal gene expression distributions is a universal feature of gene expression in eukaryots. Data sets from S. cerviciae (GSE4135), C. elegans (GSE8004), and humans (GSE8853) were examined. The distribution of log-transformed gene expression levels was described by 3-log-normal distributions in all species examined (A–D). A human sample was examined with RNAseq. Approximately 23 thousand unique tags produced 17 million sequences. The distribution of log-transformed sequence tag counts was fit by 3-log normal distributions (E).

A previous study of the large-scale organization of gene expression levels identified power-law like behavior as a universal feature of gene expression levels as measured by microarrays or serial analysis of gene expression (SAGE) [8]. Hundreds of data sets from S. Cerevisiae to humans were examined. A power law distribution occurs when the probability that a measurement will have a level, k, is proportional to *k* ^−*r*^, where ‘−r’ is a constant that represents the slope of the power law distribution in the log-log scale. The referenced study of gene expression reported power law-like behavior with an ‘r’ in the range of 1.5 to 2 [8]. Distributions are described as power law-like, instead of following a true power law, because at very high levels, the power law does not apply and the probability becomes spread out into a “fat tail.” Power law-like behavior is common in nature. Using published methods, we found that our data also was power law-like with an ‘r’ in the range of 1.7 to 2. This indicated that our findings were consistent with previous reports on the large-scale organization of gene expression levels, i.e., data that could be described by 3 log normal distributions also appeared power law-like. Because power law-like behavior is universal for gene expression levels measured by microarrays including examination our data, we hypothesized that the finding that the distributions of gene expression levels could be described by multiple log-normal distributions might also be universal. We obtained gene expression data sets from the Gene Expression Omnibus public repository. Data sets from S. Cerevisiae (GSE4135), C. Elegans (GSE8004), and humans (GSE8853) were examined. As predicted, we found that the distribution of log-transformed gene expression levels was described by 3-log-normal distributions in all species examined (figure 6).

**Figure 7:**
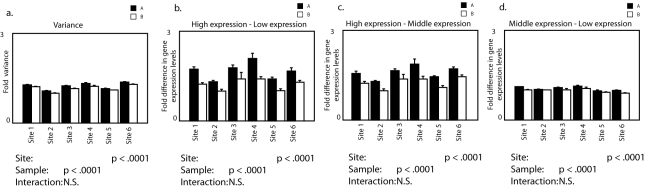
Global differences in gene expression patterns are robustly detected at multiple testing sites. To validate our approach to microarray analysis, we analyzed an independent publicly available dataset from the MicroArray Quality Control Project. Here two commercially available samples (Sample A = Stratagene Universal Human Reference RNA (UHRR, Catalog #740000), Sample B = Ambion Human Brain Reference RNA (HBRR, Catalog #6050)) were sent to six independent testing sites that ran 5 technical replicates for each sample. At all testing sites sample A had a greater variance of log-transformed gene expression levels than sample B. In all cases this was accompanied by a moving apart of gene expression distributions. A strongly significant effect of testing site on all measurements was also present. The effects of sample and site did not interact.

We confirmed the finding of multiple log-normal distributions of gene expression in humans with a data set from an RNAseq analysis (data was provided by TGen; high throughput sequencing was performed by Agencourt Bioscience Corporation, Beverly, MA). RNAseq uses high throughput sequencing to sequence an entire gene expression library. Unique tags are counted to obtain an expression level based on the number of counts. Because RNAseq directly counts the number of sequences, technical artifacts from differences in probe hybridization will not be present. The analyzed data set included approximately 17 million sequences for approximately 23 thousand unique tags. Only tags with greater than 10 sequences were used for the analysis. The distribution of gene expression data from RNAseq of a human sample had many similarities to the distribution patterns of gene expression levels as measured by Affymetrix microarrays. In both cases, distributions of gene expression levels were fit well with 3 log-normal distributions. The distributions of gene expression levels covered a similar range of gene expression levels with a range of approximately 10 natural logs for RNAseq and 11 natural logs for Affymetrix microarrays. Differences were also present. Most notably, the middle expression distribution was smaller in the RNAseq data set (figure 7). As more experiments are done with RNAseq, a clearer picture of the large-scale organization of gene expression levels may become apparent. Future experiments using RNAseq on samples like ours would provide added support for our findings with an independent method.

### Measurement of the variance in log-transformed gene expression levels reliably detects biological differences between samples

It is well recognized that hybridization conditions can affect the measurement of gene expression levels by microarrays. To determine if our method is also sensitive to the effects of hybridization conditions and whether our method can reproducibly detect biological differences in the variance in log-transformed gene expression levels across multiple experiments, we used data from the Microarray Quality Control Project (MAQC). Here two commercially available samples (Sample A = Stratagene Universal Human Reference RNA (UHRR, Catalog #740000), Sample B = Ambion Human Brain Reference RNA (HBRR, Catalog #6050)) were sent to six independent testing sites that ran 5 technical replicates on each sample. Using our approach we detected significant differences always in the same direction in the large-scale organization of gene expression levels between sample A and B at all testing sites (supplemental figure S6). Specifically, sample A always had a higher variance in log-transformed gene expression levels. This was always accompanied by an increased separation of log-normal distributions of gene expression levels. Importantly, we found independent statistical main effects on the large-scale organization of gene expression levels for biological factors associated with the sample and technical factors associated with the testing site with no statistical interaction (figure 7). This emphasizes the importance of hybridizing all samples together when microarrays are used to look for biological differences in the large-scale organization of gene expression levels. The importance of parallel processing of samples was also suggested by our finding that technical factors associated with the method of RNA processing affected the measurement of the large-scale organization of gene expression levels. In the handling experiment, we switched our method of RNA extraction from using Trizol to Qiagen RNA/DNA columns in order to have DNA for future bisulfite sequencing. All microarrays were hybridized together. Despite simultaneous hybridization there was a statistically significant effect of the RNA processing method (not shown). We found increased variance in log-transformed gene expression levels in samples that were processed with RNA/DNA columns compared to samples processed with Trizol (Handling: p<.0001, RNA processing: p<.0001, interaction: non-significant). Nonetheless, even in the presence of an effect of RNA processing method, we found significant effects related to biological factors associated with postnatal handling using either method and a statistical main effect of handling for the entire sample together (figures 2 and 5). There was no statistical interaction of RNA processing method with postnatal handling on the large-scale organization of gene expression levels. These results indicate, as is true for traditional methods of microarray analysis, that processing samples together and in an identical manner at all levels is likely to improve the detection of biological differences in the organization of gene expression levels.

## Discussion

We found in multiple microarray experiments that differences in the large-scale organization of gene expression levels in the hippocampus, rather than specific differences in gene expression levels, were associated with individual variability in hippocampus-related behaviors. We implicated epigenetically mediated effects on the large-scale organization of gene expression levels by doing experiments in genetically identical mice and by using an early developmental intervention that modifies epigenetic factors and adult mouse behavior [19], [20] to elicit changes in the large-scale organization of gene expression levels. We showed that even in outbred populations of mice that were selected for extremes of behavior, where presumably genetic effects would be operating, that the large-scale organization of gene expression levels continued to be associated with behavioral variability. This suggests that a similar association of the large-scale organization of gene expression levels with behavioral variability may be present in outbred populations such as humans and that there may be interactions between genetic and epigenetic factors in the regulation of the large-scale organization of gene expression levels. Using the Microarray Quality Control (MAQC) data set, we demonstrated that our method reliably detects biologically mediated differences in the large-scale organization of gene expression levels, but that it is also sensitive to technical factors such as hybridization batch and RNA processing method. Finally, we demonstrated that changes in the variance in log-transformed gene expression levels can be explained by the degree of separation of 3 large log-normally distributed gene expression families and that the presence of these log-normally distributed families is a universal characteristic of gene expression in eukaryotes.

Though, our results are surprising, many lines of evidence make global epigenetically mediated changes in gene expression a plausible mechanism underlying individual variability in behavior. Differences in gene expression levels and behavior that are influenced by early life experience are associated with epigenetic modifications that are reversed along with changes in behavior and gene expression through treatment with histone deacetylase inhibitors [7]. Specifically, rats that receive low levels of maternal care as pups and are less active in the open field as adults become indistinguishable from rats that receive high levels of maternal care when they are treated with with histone deacetylase inhibitors [7]. Histone deacetylase inhibitors are general inhibitors of chromatin remodeling enzymes. Global changes in chromatin remodeling, in turn, are shown to have extensive effects on gene expression in multiple contexts [21]–[23]. Histone deacetylase inhibitors have also been found to increase synaptogenesis, improve learning, and even to stimulate recovery of lost memories in a mouse model of Alzheimer disease [24]–[27]. Taken together evidence points towards the potential role of epigenetic effects on the large-scale organization of gene expression levels in establishing neuronal properties and behavior.

Our results appear to conflict with reports that suggest that the effects of epigenetic interventions on gene expression act only at the level of specific genes [19], [28]–[32]. Specific differences in gene expression, however, are identified after normalizing for global differences in the level of gene expression, thus, global differences may not be visualized in these analyses. We believe that specific differences in gene expression that are noted by others may represent genes that are most affected by changes in the large-scale organization of gene expression levels. In our model, log-normal distributions of gene expression levels represent groups of genes that respond in a similar manner to factors that regulate the large-scale organization of gene expression levels. Within these groups of genes, we predict there will be specific genes that may be primed to respond preferentially to changes in the factors that influence the large-scale organization of gene expression levels. It is these sensitive genes that would be most likely to be identified when attempts are made to find specific differences in gene expression levels. Future work is necessary to determine the relative contributions of specific gene expression levels versus global gene expression organization to the effects of epigenetic interventions on cell function, behavior, and pathology.

A further shortcoming of our study is the use by microarrays of probe hybridization as a measure of gene expression. Hybridization conditions are known to affect the measurement of specific gene expression levels and we found that there are also effects of hybridization on the measurement of the large-scale organization of gene expression levels. Despite the presence of effects of hybridization, we also showed using the MAQC data set that biologically related differences in the large-scale organization of gene expression levels are consistently and reproducibly detected. Similarly we found even in the presence of technical effects of RNA processing method that biologically related differences in the large-scale organization of gene expression levels associated with postnatal handling were detected using either method of RNA processing. A potentially less technically sensitive measurement of the large-scale organization of gene expression levels is theoretically possible with the emerging technology of RNAseq [33] in which high throughput sequencing allows the sequencing and counting of all mRNA species within a sample. We used RNAseq to confirm the presence of 3 log-normal distributions of gene expression in a human sample.

Our measurement of the large-scale organization of gene expression levels is associated with biological differences between the brains of animals separated by behavior or by postnatal experience, nonetheless, it is possible that a biological property of the brains influences the microarray measurement of gene expression levels but does not influence actual gene expression levels. We do not believe that this is the case. If differences in gene expression levels were associated with biological effects on measurement by microarray and not to actual differences in gene expression levels, then microarrays would not successfully detect gene expression changes that are confirmed by other methods such as *in situ* hybridization and real-time PCR. Supporting this, the research group studying high/low freezing mice used real-time PCR to validate 6 candidate genes in their study [14]. This indicates that traditional gene-specific approaches to microarray analysis need not exclude examination of the large-scale organization of gene expression levels and that the two approaches are likely to compliment each other in the understanding of gene expression correlates of biological processes.

Our experiments suggest substantial variability in the large-scale organization of gene expression levels within the hippocampus. For instance when the low expression group of genes is used as a reference, the expression levels of high expression genes within handled or high activity mice were increased on average by greater than 1.5-fold. The increase in the expression level of genes in the high expression group relative to genes in the low expression group suggests a differential allocation in resources for gene expression. This is seen as a skewing of gene expression resources in more active mice towards high expression genes and away from low expression genes. These large-scale differences may not be readily detected when gene expression is normalized to overall signal on a microarray or examined by real-time PCR because, in both cases, normalization is relative to gene expression within the group of high expression genes. It must be noted that the true expression level differences between groups of genes may be greater or less than those determined by microarrays. This is because, as previously discussed, microarrays use hybridization signal to estimate relative expression levels and do not directly count the number of mRNAs present. Analysis of a human sample with RNAseq, however, demonstrated a range of log-transformed gene expression levels that was similar to the range of expression levels found using microarrays. The fact that both methods detect similar ranges of expression levels increases confidence in the accuracy our measurements.

At this point, the biological meaning of a skewing of gene expression levels is unclear. One interesting possibility is that skewing of gene expression levels is related to the level of cellular maturation in the brain regions that were examined. As cells differentiate and mature there is a tendency of gene expression to become more specialized and thus skewed towards genes that are important to a cell's specific function. The interrelationship between epigenetic factors and cellular maturation is well established within the field of oncology [34]. Within the field of neuroscience, the interrelationship between epigenetic factors and neuronal maturation and plasticity is increasingly recognized and studied. HDAC inhibitors can increase memory formation and synaptogenesis [24]–[27]. Removal of MECP2, a methylated DNA binding protein that is causally related to the autism spectrum disorder Rett Syndrome, leads to pronounced deficits in neuronal maturation and dendritic spine density and spine maturation [35], [36]. Again it can be argued that each of these effects is related to specific and not to global changes in gene expression levels. We cannot, at this point, rule out this possibility and, moreover, it is possible, that even in the context of the global changes that we find, only a limited number of genes are important for the differences in behavior that we observe. Even if it is true that only a few genes are relevant to the differences in behavior that we find, our results indicate, under the conditions studied, that the mechanism leading to the large-scale differences in gene expression is resulting in a previously unrecognized global level of change. We hope that the recognition of this global level of gene expression organization will encourage future studies to understand the potential functional relevance of the large-scale organization of gene expression levels to brain development, pathology, and treatment.

## Materials and Methods

### Animals

Female Balb c/J mice were used for open field activity and handling experiments. Mice were group housed and maintained on a 12-h/12-h light/dark cycle with food and water available ad libitum were used for all experiments. All animal protocols were reviewed and approved as meeting appropriate ethical standards by Columbia University's and New York State Psychiatric Institute's IACUC boards.

### Postnatal Handling

On postnatal days 1–14 pups were separated from mothers for 15 minutes each morning. Pups and mothers were placed in clean cages during this period and then returned to their original cages. For microarray array experiments (GSE11679) we used all 25 females from 11 litters (5 handled and 6 non-handled). There were a total of 11 handled females and 14 non-handled females. 4 animals (2 handled and 2 non-handled) were excluded from open-field behavioral analyses secondary to equipment malfunction on testing day 2.

### Open field behavior

Activity in an open field is quantified in four Plexiglas open field boxes 43×43 cm^2^ with two sets of 16 pulse-modulated infrared photobeams (MED Associates).

### Light-dark behavior

A light-dark partition (MED Associates) was inserted into an open field box. Because of high anxiety-like behavior we start Balb c/J mice in the dark arena and allow animals 30 minutes exploration time. Activity is quantified as above for open field in four Plexiglas open field boxes 43×43 cm^2^ with light-dark partitions dividing the arena equally in half.

### Composite Behavioral Score

Composite behavior represents average mean standardized scores across 5 open field behavioral measure (total ambulation, ambulation in center, percent ambulation in center, time in center, and entries into center) over multiple testing sessions. In the handling experiment, 4 animals (2 handled and 2 non-handled) were excluded from the calculation of behavioral scores secondary to equipment malfunction.

### Tissue collection

Whole hippocampus was removed from sacrificed animals and place in RNAlater. The CA1 region was micro-dissected from 500 micron vibratome slices and placed in RNAlater.

### Expression profiling

Total RNA was extracted using Qiagen RNA/DNA columns or Trizol, double round amplified, cleaned, and biotin-labeled, and fragmented using standard Affymetrix protocols. Labeled RNA was hybridized to an Affymetrix Mouuse 430_2.0 Array scanned on Affymetrix's GeneChip Scanner 3000 7G and reviewed for quality control.

### Digital Gene Expression (RNAseq)

One microgram of total RNA was used to create a Mme1 expression tag library for SOLiD sequencing. cDNA was synthesized on a solid support (Dynabead oligo (dT_25_)) using the Superscript cDNA synthesis kit (Invitrogen) and the mRNA direct kit (Invitrogen, ) according to manufacturers protocols. cDNA's were digested with NLA III to anchor the sequence tag to the 3′most NLA III site, after which a tagging adaptor containing an Mme1 recogniton site, and a SOLiD system PCR adaptor sequence, was ligated to the bead-attached cDNA. This allowed for the creation of a 20 bp sequence tag. A second PCR adaptor was ligated to the adaptor/tag. The final product was amplified with 11 cycles prior to emulsion PCR and sequencing on the SOLiD sequencing platform.

### Microarray data analysis

Affymetrix .cel files were imported into Affymetrix Expression Console version 1.1. Data was pre-processed and summarized using Microarray Analysis Suite (MAS) 5.0. MAS 5.0 is used for data processing because it lacks a distribution normalization step. RMA, Plier, and other commonly used processing methods introduce a quantile normalization step that may minimize biological differences in gene expression distribution patterns. Other labs have reported the benefits of using MAS 5.0 in global assessments of gene expression e.g. genetic co-regulatory networks [37].

### Microarray data deposition

Microarray data has been deposited in the Gene Expression Omnibus public repository (http://www.ncbi.nlm.nih.gov/geo/). The accession number for the high-low open field activity data set is GSE11680. The accession number for the handling experiment data set is GSE11679.

### Data sets

We analyzed microarray data sets GSE4034 and GSE4035 from the Gene Expression Omnibus public repository. Affymetrix .cel files were downloaded from this site and analyzed as described above. For validation of our technique we downloaded data from GSE5350 (Microarray Quality Control Project). For evaluation of distribution of gene expression in other species we downloaded GSE4135 (S. Cerviciae), GSE8004 (C. Elegans), and GSE8853 (H. Sapiens).

### Log-normal distributions

Log transformed data was standardized by dividing by the mean, and frequency was determined by distributing the data into 50 equally sized bins. 25 bins were used for intra-species comparisons and RNAseq because of a smaller number of probe sets on some microarray platform and a smaller number of unique tags using RNAseq compared to probe sets on the Mouse 430_2.0 microarray. Data is initially handfit to a distribution to obtain starting parameters with an error less than 10. Fit error is calculated as the variance standardized to the number of probe sets on a microarray. Best-fit parameters are then calculated using the Matlab Optimization Toolbox (The Mathworks, 2007). Three log-normal distributions visually appear to be the best fit. We calculated fit error for 1, 2, 3, and 4 Gaussian models and found that three Gaussians was the optimal fit. A four Gaussian model did not improve the fit (supplemental figure 1). Matlab .m files are available on request.

### Fold change in expression level of log-normal distributions

To calculate fold difference in relative expression levels of log-normal distributions, log scale differences are converted to linear fold differences. This is done by taking the natural exponent to the power of the difference between measures in the natural log scale. For instance, if the difference in the distance between high and low expression groups in handled vs. non-handled mice was 1, then the fold difference would be the natural exponent (2.718) taken to the power of 1, i.e. 2.718.

## Supporting Information

Figure S1 Stable behavioral differences are present in female Balb c/J mice over time and lighting conditions. Bar graph highlights composite behavioral scores on which groups were selected. Line graphs are shown for five open field measures (center ambulation, percent ambulation center, time in center, center entries, and total ambulation) over the course of five testing sessions on days 1, 3, 5, 33, and 35. Mice were obtained from Jackson. All mice were born on the same day. Testing began at approximately 8 weeks of age. Lighting conditions were varied between lights on (light - 200 lux) and lights off (dark). Animals were separated based on composite behavioral scores based on the average mean standardized behavioral scores for all measures and testing days. Figure demonstrates that animals separated by composite behavior are stably different in all open-field measures over time and lighting conditions, i.e. composite behavior is not biased toward a specific open-field measure.
(1.53 MB EPS)

Figure S2 Stable behavioral differences are present in female Balb c/J mice subjected to postnatal handling. Line graphs are shown for five open field measures (center ambulation, percent ambulation center, time in center, center entries, and total ambulation) over the course of three testing sessions on days 1, 3, and 5. Testing began at approximately 8 weeks of age. Figure demonstrates that handled mice are significantly more exploratory by composite behavior and individual open-field measures. 4 mice (2 handled and 2 non-handled were excluded secondary to equipment malfunction on testing day 2.
(1.19 MB EPS)

Figure S3 Behavioral differences in the light-dark paradigm are present in female Balb c/J mice subjected to postnatal handling. Bar graphs are shown for five open light-dark measures (light ambulation, percent ambulation light, time in light, light entries, and total ambulation). Testing was conducted at approximately 9 weeks of age. Figure demonstrates that handled mice are significantly more exploratory in the light but do not exhibit an overall increase in total ambulation.
(0.91 MB EPS)

Figure S4 Outbreeding strategy used for selection on contextual fear. Figure demonstrates breeding strategy used by researchers from which microarray data sets were obtained via public repository [14]. An anxious strain, DBA or A/J, and a non-anxious strain,C57/B6 ,were crossed to obtain an F1 generation that was intercrossed to obtain F2. F2 was selected based on contextual freezing. The top and bottom approximately 10% were used to generate the next generation which was continued for 4–5 generations.
(1.14 MB EPS)

Figure S5 Global genomic properties in hippocampus but not amygdala are associated with conditioned fear, open field exploration, elevated plus behaviors. Replicate experiment of that described in figure 4. Mice for these experiments were selected over multiple generations after an inter-strain cross of a high (DBA) and low (C57/B6) strain of mice. Using the same methods as the previous experiments we find that global gene expression patterns in the hippocampus but not amygdala are significantly different between high and low freezing mice. In this study low freezing mice were found to have increased exploratory behavior in the open-field similar to high exploratory and handled mice [14].
(1.30 MB EPS)

Figure S6 Matlab curve fitting program works across a wide range of global gene expression patterns. Panel (a) shows the mouse from the handling experiment with the greatest overall variance, while panel (b) shows the mouse with the least variance. Panel (c) shows the overlap. Figure demonstrates that at the extremes curve fits are excellent. Similar fits were obtained for all intermediate distributions.
(0.73 MB EPS)

## References

[1] Wong AH, Gottesman, Petronis A (2005). Phenotypic differences in genetically identical organisms: the epigenetic perspective.. Hum Mol Genet.

[2] Petronis A (2006). Epigenetics and twins: three variations on the theme.. Trends Genet.

[3] Kendler KS, Halberstadt LJ, Butera F, Myers J, Bouchard T (2007). The similiarity of facial expressions in response to emotion-inducing films in reared-apart twins.. Psychol Med.

[4] Bouchard TJ, Lykken DT, McGue M, Segal NL, Tellegen A (1990). Sources of human psychological differences: the Minnesota Study of Twins Reared Apart.. Science.

[5] Gartner K (1990). A third component causing random variability beside environment and genotype. A reason for the limited success of a 30 year long effort to standardize laboratory animals?. Lab Anim.

[6] Weaver IC, Szyf M, Meaney MJ (2002). From maternal care to gene expression: DNA methylation and the maternal programming of stress responses.. Endocr Res.

[7] Weaver IC, Champagne FA, Brown SE, Dymov S, Sharma S (2005). Reversal of maternal programming of stress responses in adult offspring through methyl supplementation: altering epigenetic marking later in life.. J Neurosci.

[8] Ueda HR, Hayashi S, Matsuyama S, Yomo T, Hashimoto S (2004). Universality and flexibility in gene expression from bacteria to human.. Proc Natl Acad Sci U S A.

[9] Kuznetsov VA, Knott GD, Bonner RF (2002). General statistics of stochastic process of gene expression in eukaryotic cells.. Genetics.

[10] Ray J, Hansen S (2005). Temperamental development in the rat: the first year.. Dev Psychobiol.

[11] Kazlauckas V, Schuh J, Dall'Igna OP, Pereira GS, Bonan CD (2005). Behavioral and cognitive profile of mice with high and low exploratory phenotypes.. Behav Brain Res.

[12] Meaney MJ, Mitchell JB, Aitken DH, Bhatnagar S, Bodnoff SR (1991). The effects of neonatal handling on the development of the adrenocortical response to stress: implications for neuropathology and cognitive deficits in later life.. Psychoneuroendocrinology.

[13] Liu D, Diorio J, Day JC, Francis DD, Meaney MJ (2000). Maternal care, hippocampal synaptogenesis and cognitive development in rats.. Nat Neurosci.

[14] Ponder CA, Kliethermes CL, Drew MR, Muller J, Das K (2007). Selection for contextual fear conditioning affects anxiety-like behaviors and gene expression.. Genes Brain Behav.

[15] Ponder CA, Huded CP, Munoz MB, Gulden FO, Gilliam TC (2008). Rapid selection response for contextual fear conditioning in a cross between C57BL/6J and A/J: behavioral, QTL and gene expression analysis.. Behav Genet.

[16] Bishop JO, Morton JG, Rosbash M, Richardson M (1974). Three abundance classes in HeLa cell messenger RNA.. Nature.

[17] Axel R, Feigelson P, Schutz G (1976). Analysis of the complexity and diversity of mRNA from chicken liver and oviduct.. Cell.

[18] Limpert E, Stahel W, Abbt M (2001). Log-normal distributions across the sciences: keys and clues.. Bioscence.

[19] Weaver IC, Meaney MJ, Szyf M (2006). Maternal care effects on the hippocampal transcriptome and anxiety-mediated behaviors in the offspring that are reversible in adulthood.. Proc Natl Acad Sci U S A.

[20] Weaver IC, Cervoni N, Champagne FA, D'Alessio AC, Sharma S (2004). Epigenetic programming by maternal behavior.. Nat Neurosci.

[21] Li B, Carey M, Workman JL (2007). The role of chromatin during transcription.. Cell.

[22] Shen S, Li J, Casaccia-Bonnefil P (2005). Histone modifications affect timing of oligodendrocyte progenitor differentiation in the developing rat brain.. J Cell Biol.

[23] Tao R, de Zoeten EF, Ozkaynak E, Chen C, Wang L (2007). Deacetylase inhibition promotes the generation and function of regulatory T cells.. Nat Med.

[24] Lattal KM, Barrett RM, Wood MA (2007). Systemic or intrahippocampal delivery of histone deacetylase inhibitors facilitates fear extinction.. Behav Neurosci.

[25] Fischer A, Sananbenesi F, Wang X, Dobbin M, Tsai LH (2007). Recovery of learning and memory is associated with chromatin remodelling.. Nature.

[26] Levenson JM, O'Riordan KJ, Brown KD, Trinh MA, Molfese DL (2004). Regulation of histone acetylation during memory formation in the hippocampus.. J Biol Chem.

[27] Vecsey CG, Hawk JD, Lattal KM, Stein JM, Fabian SA (2007). Histone deacetylase inhibitors enhance memory and synaptic plasticity via CREB:CBP-dependent transcriptional activation.. J Neurosci.

[28] Weaver IC, La Plante P, Weaver S, Parent A, Sharma S (2001). Early environmental regulation of hippocampal glucocorticoid receptor gene expression: characterization of intracellular mediators and potential genomic target sites.. Mol Cell Endocrinol.

[29] Weaver IC, Diorio J, Seckl JR, Szyf M, Meaney MJ (2004). Early environmental regulation of hippocampal glucocorticoid receptor gene expression: characterization of intracellular mediators and potential genomic target sites.. Ann N Y Acad Sci.

[30] Meaney MJ, Aitken DH, Bodnoff SR, Iny LJ, Tatarewicz JE (1985). Early postnatal handling alters glucocorticoid receptor concentrations in selected brain regions.. Behav Neurosci.

[31] Liu D, Diorio J, Tannenbaum B, Caldji C, Francis D (1997). Maternal care, hippocampal glucocorticoid receptors, and hypothalamic-pituitary-adrenal responses to stress.. Science.

[32] Tsankova N, Renthal W, Kumar A, Nestler EJ (2007). Epigenetic regulation in psychiatric disorders.. Nat Rev Neurosci.

[33] Mortazavi A, Williams BA, McCue K, Schaeffer L, Wold B (2008). Mapping and quantifying mammalian transcriptomes by RNA-Seq.. Nat Methods.

[34] Mulero-Navarro S, Esteller M (2008). Epigenetic biomarkers for human cancer: The time is now.. Crit Rev Oncol Hematol.

[35] Smrt RD, Eaves-Egenes J, Barkho BZ, Santistevan NJ, Zhao C (2007). Mecp2 deficiency leads to delayed maturation and altered gene expression in hippocampal neurons.. Neurobiol Dis.

[36] Zhou Z, Hong EJ, Cohen S, Zhao WN, Ho HY (2006). Brain-specific phosphorylation of MeCP2 regulates activity-dependent Bdnf transcription, dendritic growth, and spine maturation.. Neuron.

[37] Lim WK, Wang K, Lefebvre C, Califano A (2007). Comparative analysis of microarray normalization procedures: effects on reverse engineering gene networks.. Bioinformatics.

